# Clustering and Heterogeneous P1 Distributions in Diamond Govern DNP Mechanisms at 6.9 and 13.8 T

**DOI:** 10.1021/acs.jpclett.5c01809

**Published:** 2025-10-13

**Authors:** Orit Nir-Arad, David H. Shlomi, Raj K. Chaklashiya, Nurit Manukovsky, Ilia Kaminker

**Affiliations:** † School of Chemistry, Faculty of Exact Sciences, 26745 Tel Aviv University, Tel Aviv 6997801, Israel; ‡ Department of Chemistry, Northwestern University, 633 Clark Street, Evanston, Illinois 60208, United States; § Materials Department, 8786 University of California, Santa Barbara, Santa Barbara, California 93106, United States

## Abstract

Nitrogen substitutions (P1 centers) in diamonds were shown to provide significant ^13^C hyperpolarization at room temperature and high fields, with the presence of P1 clusters hypothesized to be essential. We compare the 6.9 and 13.8 T ^13^C dynamic nuclear polarization (DNP) and electron paramagnetic resonance spectra of two single-crystal diamonds: one with a high P1 concentration (∼20 ppm) and abundant P1 clusters, and another with a low P1 concentration (∼3.5 ppm) and no clusters. The first exhibits a 540 ± 180 nm ^13^C NMR enhancement with broad and narrow DNP features, whereas the second displays only weak, sharp DNP peaks after prolonged irradiation. The dependence of ^13^C polarization buildup rate on millimeter-wave frequency distinguishes solid-effect (SE) from cross-effect (CE), confirming P1 clusters are essential for efficient high-field DNP. Moreover, we show that in the high-P1-concentration diamond, the SE and CE polarize distinct pools of ^13^C nuclei, demonstrating DNP’s ability to map the spatial distribution of diamond defects.

## Graphical abstract

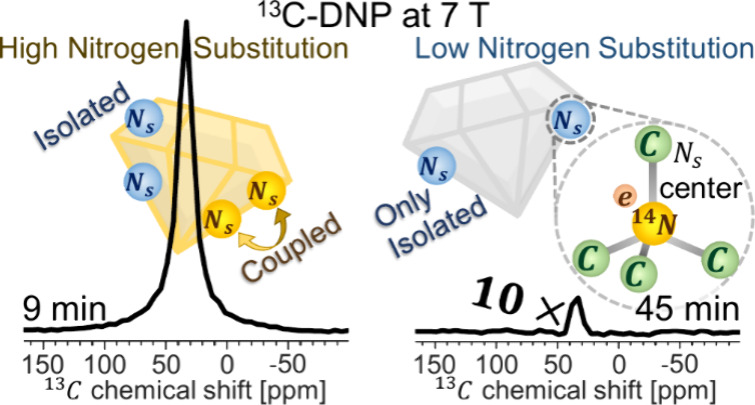

Dynamic nuclear polarization (DNP) can enhance nuclear magnetic resonance (NMR) signals by orders of magnitude, vastly expanding the range of NMR applications in a variety of fields, ranging from structural biology to material science.^1–4^ At the center of the DNP experiments lies the polarization transfer from unpaired electron spins to the nuclear spins of interest. DNP efficiency is thus largely dependent on the paramagnetic center used, its atomic environment, and the electron spin dynamics. The latter information can only be discerned via electron paramagnetic resonance (EPR) experiments, ideally performed at the same temperature and magnetic field strength as the DNP experiment. This means magnetic fields of ≥ 7 T and temperatures of ≥ 100 K, as in most solid-state (ss) NMR experiments conducted nowadays.

Paramagnetic defects in diamonds were shown to provide large DNP enhancements at room temperature; they serve as an excellent model system for the investigation of a complex interplay of DNP mechanisms^5–11^ and were recently shown to be useful for the hyperpolarization of biomolecules.^12^ Nitrogen substitution centers, also termed P1 centers, are the most common nitrogen-based defects in diamonds.^13^ They occur when a single nitrogen atom substitutes a carbon in the diamond lattice, resulting in an unpaired electron with spin 1/2. P1 centers have been investigated for decades in EPR experiments, with most studies performed at relatively low magnetic fields (≤3 T).^14–20^ Unlike other ss-DNP polarizing agents, P1 centers are characterized by very long relaxation times (T_1_ in the millisecond range and *T*_m_ in the microsecond range at room temperature), which makes them suitable for room temperature DNP.

Our group and the group of Songi Han and collaborators recently showed that a substantial amount of P1 centers (∼40%) in high-pressure high-temperature (HPHT) diamonds exist as clusters with strong dipolar and/or exchange couplings.^7,8^ This clustered population was hypothesized to be essential for the efficient P1-DNP enhancement observed at high magnetic fields. This is not the only case of this kind, and clusters were proposed to play an important role in DNP in other systems as well, such as TetraTrityl,^21^ a trityl-based tetra-radical, and sulfonated BDPA.^22^

In order to conclusively prove that the clustered P1 centers play an essential role in P1-DNP and to understand the relevant DNP mechanisms by which they facilitate the nuclear hyperpolarization, we herein expand our investigation of P1-DNP at 6.9 and 13.8 T.^8^ We compare two single-crystal diamond samples, with high and low P1 concentration, and identify the DNP mechanisms mediated by each P1 population using a combined analysis of EPR and ^13^C-DNP results of each sample. This was achieved by utilizing the capability of our group’s dual DNP/EPR instrument to measure both EPR and ^13^C-DNP spectra of the same sample under identical conditions.^23^

We start by comparing the EPR spectra of the two diamonds. Unlike many EPR works on P1 centers, we chose to use single-crystal diamonds rather than diamond powders, as the EPR line shape of the former facilitates the identification of P1 clusters. As we previously reported, the P1 cluster signal is manifested as a broad, featureless signal between the sharp, well-resolved lines of the isolated P1 centers in the single crystal. High-field EPR measurements allow for the spectral separation of P1 centers from other paramagnetic centers, allowing for the identification of P1 clusters. EPR simulations allow to quantify the relative contribution of each P1 population,^8^ as was recently used by our group and others for both single-crystal diamond and microdiamond powder.^8,9,24,11^

As we previously reported,^8^ the EPR spectrum of an HPHT diamond with a P1 concentration of ∼ 20 ppm, which will be termed concentrated diamond in the rest of the text, consists of sharp signals belonging to the isolated P1 centers overlaid with a broad signal from the P1 clusters, as shown in the echo detected (ED) EPR spectrum, acquired at 6.9 T and room temperature, in black in Figure 1a. The pulse sequence used is shown in the inset. The broad signal, which accounts for 40% or 8 ppm of P1 centers, is the result of a combined contribution of P1 pairs with dipolar interaction of 3.3–50 MHz, corresponding to an interspin distances of 1–2.5 nm, and P1 clusters of two or more strongly coupled P1 centers with both exchange and dipolar interactions (J of 110–200 MHz), comparable to sub-1 nm interspin distances. The combination of these two contributions is shown in purple in Figure 1b, and we will refer to it as coupled P1 centers or P1 clusters. The decomposition of the simulation into the isolated and coupled P1 populations is shown below in light blue and purple, respectively (Figure 1b), and the combined simulation in pink is overlaid with the experimental data in Figure 1a. The full simulation parameters are detailed in the Supporting Information. The difference in intensity between the *m*_*I*_ = ± 1 manifolds observed in the experimental spectrum and absent in the simulation is the result of output power variation with frequency and the presence of standing waves in the quasi-optical system.

**Figure 1:**
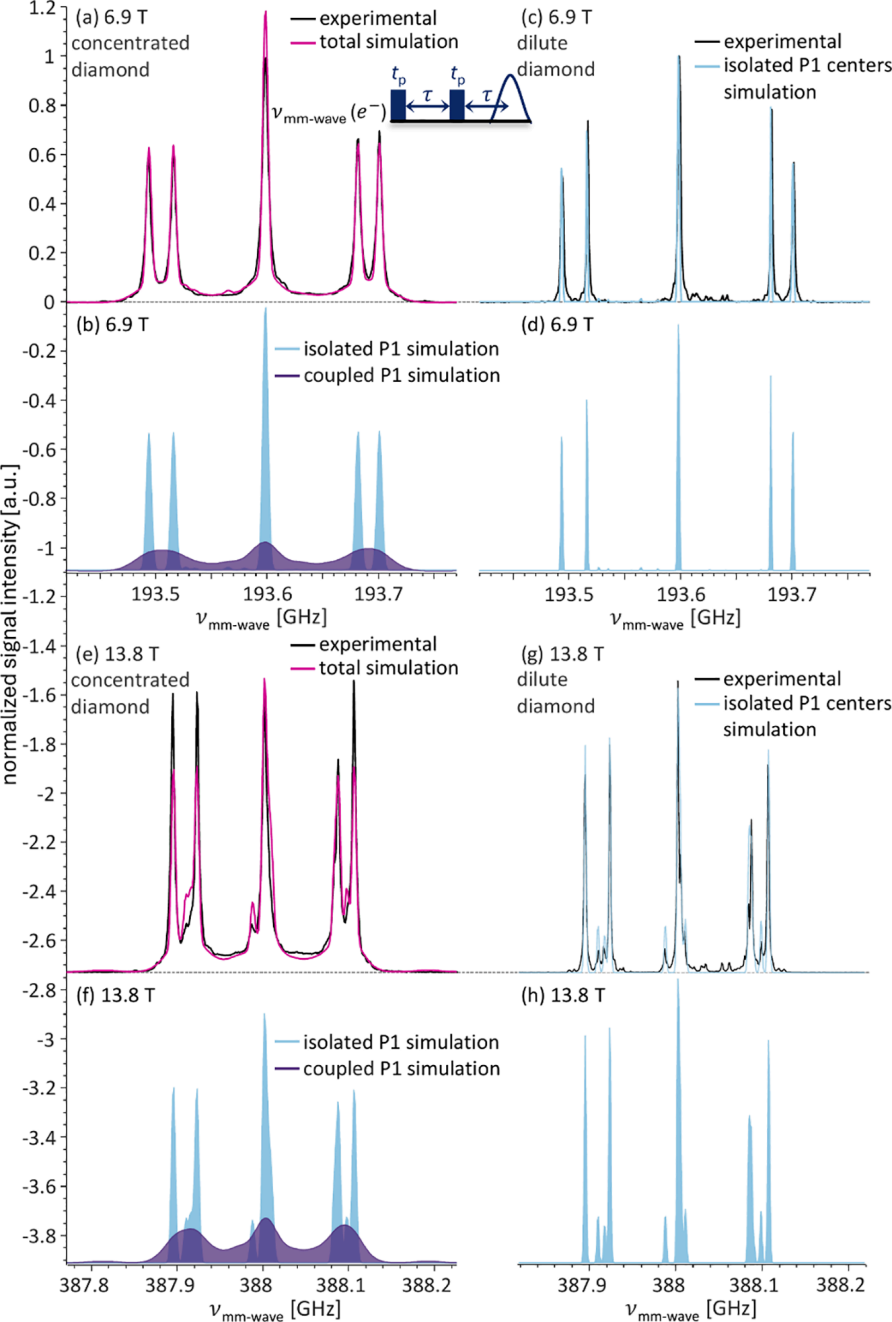
Overlay of experimental and simulated ED–EPR spectra of P1 centers in the (a and e) concentrated and (c and g) dilute diamonds acquired at (a and c) 6.9 T and (e and g) 13.8 T and room temperature. The decomposition of the simulated spectrum into the contributions of each P1 population is shown below.

In stark contrast, the broad signal is absent in the second sample, a chemical vapor deposition (CVD) single-crystal diamond with a low P1 concentration of ∼ 3.5 ppm, which will be termed dilute diamond in the rest of the text (shown in Figure 1c). We, therefore, conclude that there is no appreciable amount of coupled P1 centers present in the dilute diamond sample. This is also reinforced by the ED-EPR simulation, which requires only a single component with no dipolar or exchange interaction and a smaller line width to reproduce the experimental spectrum (Figure 1d).

The same observation remains true when comparing the ED-EPR spectra and simulations of both diamonds at 13.8 T, shown in Figures 1e-h. The difference in line shape between the 6.9 and 13.8 T ED-EPR spectra is the result of fulfilling the cancellation condition at 13.8 T, which leads to state mixing in the +1/2 electron spin manifold.^24^

We now turn to discussing the DNP spectra expected for each of the diamonds if the solid effect (SE), the simplest DNP mechanism, were the only DNP mechanism at play. We then compare these predictions with the experimental results obtained for each of the two diamonds. This comparison makes it clear that while SE is the only active DNP mechanism in the dilute diamond, this is not the case for the concentrated one.

The SE requires an electron spin and a nuclear spin coupled to each other, and is driven by irradiation on the forbidden zero-quantum (ZQ) and double-quantum (DQ) transitions.^25^ Thus, the spectral positions of the SE-DNP enhancement are shifted by the nuclear Larmor frequency away from the EPR resonance *ν*_*e*_, with a positive and negative enhancement appearing at *ν*_*e*_ - ν_13*C*_ and *ν*_*e*_ + ν_13*C*_, respectively. In addition, the SE DNP line shape reflects the EPR line shape; thus, we would expect the SE-DNP of the isolated population to manifest as sharp features, whereas the SE-DNP of the coupled population to manifest as broad features in the DNP spectrum. This allows for the assignment of the different features in the DNP spectrum, even without explicit simulations. Predicted positions of the SE DNP lines from the isolated and coupled populations at both fields are shown in Figure S1 in the Supporting Information.

We now turn to comparing those predictions with the experimental results. The ^13^C DNP sweeps (the enhanced NMR signal intensity recorded as a function of the mm-wave frequency) are shown in pink in Figures 2a and c for the concentrated diamond acquired at 6.9 and 13.8 T, respectively, and 2b and d for the dilute diamond at the same fields. All the DNP spectra are overlaid with the ED-EPR spectra in dark blue, acquired for the same diamond at the same field.

**Figure 2:**
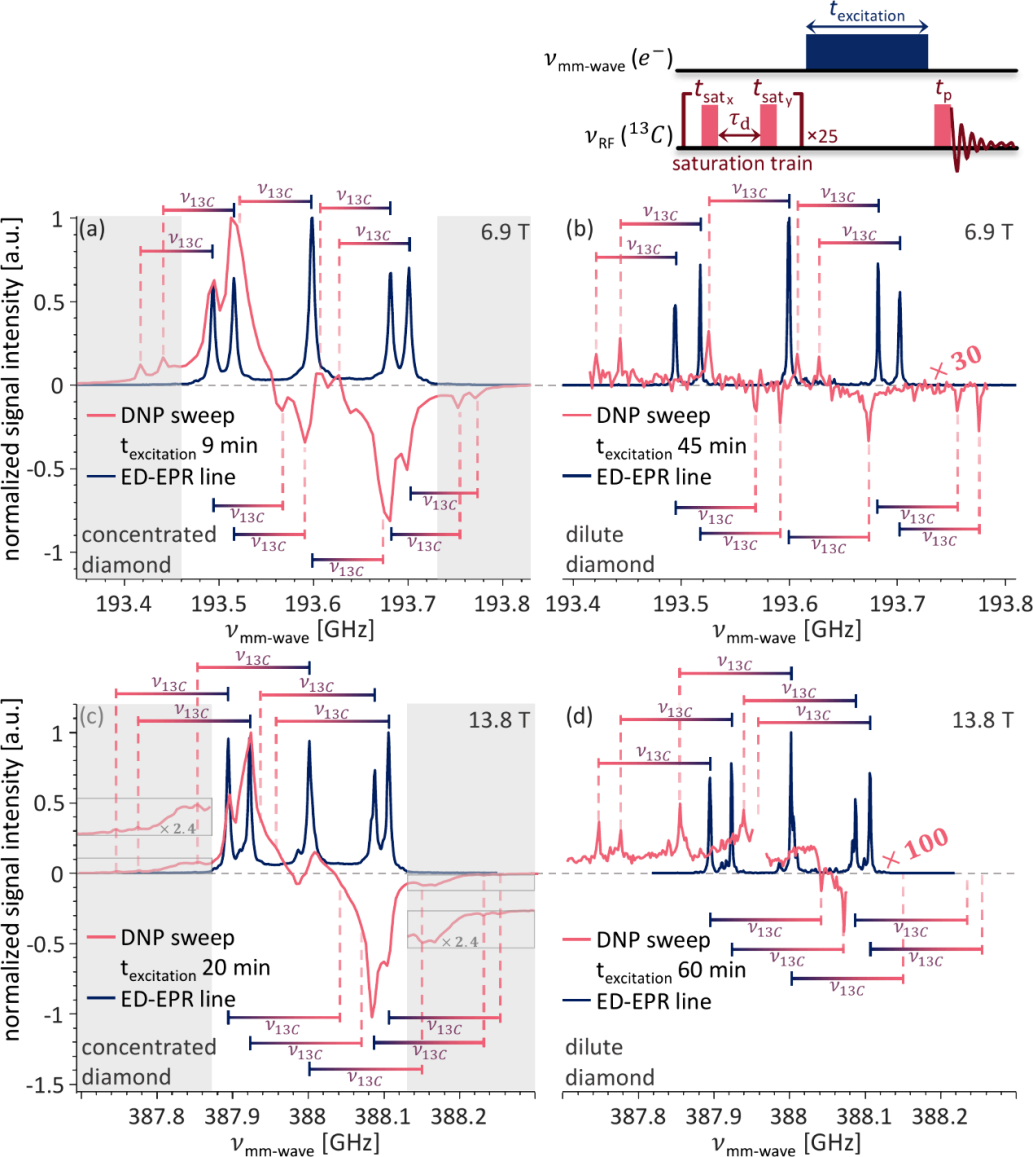
Overlays of the ED-EPR spectrum, in dark blue, and ^13^C P1-DNP sweep, in pink, of the (a and c) concentrated and (b and d) dilute diamond samples acquired at (a and b) 6.9 T and (c and d) 13.8 T and room temperature. The ^13^C P1-DNP spectra are normalized to the signal intensity of the concentrated diamond. The expected positions for the SE, calculated by shifting the ED-EPR spectra by ± the ^13^C Larmor frequency, are marked with bars, with the blue side indicating the EPR position and the pink indicating the expected SE-DNP position. The pulse sequence used for the acquisition of the ^13^C P1-DNP sweep is shown on top of the figure.

The first noticeable difference between the DNP sweeps of each sample is the signal-to-noise ratio of each spectrum. Note that the DNP spectra of both samples are normalized to the maximal signal intensity of the concentrated diamond at each field. In addition, the DNP spectra of the dilute diamond are multiplied by 30 and 100 at 6.9 and 13.8 T, respectively. The DNP sweep of the concentrated diamond (Figure 2a and c) appears noiseless, with an enhancement factor of 540 ± 180 at 6.9 T (with 9 min of irradiation). This DNP spectrum is composed of overlapping narrow and broad features. In contrast, the DNP sweeps of the dilute diamond (Figure 2b and d) are very noisy with a few sharp peaks. The maximum signal intensity in the DNP sweep of the dilute diamond is a factor of 90 and 220 lower compared to the concentrated one, at 6.9 and 13.8 T, respectively. When taking into account the 2.3 size difference of the diamonds, the difference is reduced to 39 and 96, which is unaccounted for. The signal of the thermally polarized ^13^C spectra of the dilute diamond here is prohibitively low and is therefore unknown. According to the size of the diamond samples, the signal of the dilute diamond should be ∼ 2.5 times lower than that of the concentrated one (line width effects should partially compensate for the lower signal, but are not considered), which alone cannot explain the observed difference between the diamonds. The difference becomes even more striking if one considers that the irradiation time was 45 and 60 min per point for the DNP sweeps of the dilute diamond at 6.9 and 13.8 T, respectively, compared to only 9 and 20 min for the concentrated one at 6.9 and 13.8 T. As DNP efficiency is the amount of NMR signal per unit time, these differences conclusively prove that P1-DNP at the high fields of 6.9 and 13.8 T is inefficient in the absence of coupled P1 centers.

The second difference between the DNP sweeps of both samples is their line shape. While only sharp, narrow peaks are present in the DNP sweep of the dilute diamond (Figure 2b and d), the DNP spectra of the concentrated diamond (Figure 2a and c) are much richer. For example, the maximum DNP enhancement is observed away from the position where the SE peaks are predicted. From these observations, we conclude that the DNP proceeds via different mechanisms in the two diamonds.

The DNP mechanism in place for the dilute diamond is easy to assign. Since the dilute diamond contains only isolated P1 centers, the only possible DNP mechanism is the SE. Indeed, the observed peaks in its DNP spectra all match the positions predicted in Figures S1e and j, for 6.9 and 13.8 T, respectively. They are marked with blue-pink bars in Figure 2 with colors matching the ED-EPR and DNP spectra, respectively, to aid with the peak assignment. The positive and negative enhancement positions are indicated by the relative positions of the bars above and below the spectra, respectively, on the vertical axis.

Since no DNP enhancement was observed on-resonance with the EPR transitions for the dilute diamond, which is a signature of other DNP mechanisms, we conclude that the SE is the only mechanism present in a sample devoid of P1 clusters. This explains the observed low DNP efficiency, since the SE efficiency is inversely proportional to the square of the external magnetic field strength, due to its requirement of driving the forbidden transitions, and should result in low efficiency at magnetic fields ≥ 7 T. The results obtained at 13.8 T, where 60 min, instead of 45 min at 6.9 T, of irradiation on the electron spins were required to observe a ^13^C NMR signal above the background noise, also reinforce this conclusion (Figure 2d). Due to the prolonged irradiation time of 60 min per point, acquisition of less than half of the DNP spectrum required more than a week, making acquisition of the full DNP spectrum impractical.

The sheer complexity of the DNP spectrum of the concentrated diamond (Figures 2a and c) suggests that multiple DNP mechanisms are simultaneously at play. This is not surprising and was reported previously for microdiamond powders of HPHT diamonds at 3.3,^6,7,10^ 7 T,^7,10^ and 14 T^11^ and for a single-crystal diamond at 3.3 T.^6^

The most relevant mechanism for the concentrated diamond is the cross effect (CE), which requires two coupled electron spins with the resonance frequencies *ν*_*e*__1_ and *ν*_*e*__2_, and a nuclear spin coupled to at least one of the electrons with the Larmor frequencies of the three spins fulfilling the CE condition, |*ν*_*e*__1_ - ν_*e*2_| = ν_13*C*_.^26^ For a sample whose line width does not depend on g-anisotropy, like P1 centers, the CE efficiency does not depend on the field strength.^26^ This can explain P1-DNP’s high efficiency at the high fields of 6.9 and 13.8 T. CE requires an on-resonance irradiation on one of the participating electron spins, *ν*_*e*__1_ or *ν*_*e*__2_, with a positive and a negative enhancement separated by ν_13*C*_.

The CE could occur indirectly with (partial) saturation of one of the electron spins fulfilling the CE condition occurring by propagation of excitation through cross-talk between different electron spins, via electron–electron spectral diffusion (eSD).^27,28^ This mechanism is termed indirect CE (iCE).

If one of the electron spins participating in the CE has a faster relaxation time, negating the ability to saturate it, the CE characteristic shape will be truncated (tCE), where the positive and negative enhancements no longer have the same intensity, and at the extreme, only one of the lobes remains visible.^29^

Another DNP mechanism that might be efficient at high magnetic fields is the Overhauser effect (OE), which relies on an imbalance between the ZQ and DQ relaxation rates and requires on-resonance irradiation.^30–32^ It is, however, unlikely to be involved in the DNP of an insulating solid with highly localized defects such as diamond. In addition, the Overhauser effect will result in either a positive or a negative DNP enhancement, depending on the type of the electron–nuclear coupling, but not the DNP spectra with positive and negative lobes as observed here.^33^ This assignment is in contrast to earlier reports made before the experimental observation of the coupled P1 population, which had to include the OE to account for the observed enhancement.^6^ Recent publications exclude the OE^9,11^ or claim it is unlikely to occur^7^ based on similar assertions.

In the concentrated diamond case, the maximum DNP enhancement is on-resonance with the EPR transitions, so the SE mechanism is not the main contributor in this case. We will return to the role SE plays in the concentrated diamond sample later. Thus, the main contribution in this case comes from CE or its variants tCE and iCE. For any of the CE family of DNP mechanisms, we expect a much faster buildup of hyperpolarization compared to SE. This is indeed the case, with the DNP buildup time *t*_*buildup*_ being 3.8 min with a stretching factor *β*_*buildup*_ of 0.8 (using a stretched exponential model $e^{-{\left(\frac{x}{t_{buildup}}\right)}^{\beta_{buildup}}}$ ) at the maximum of the DNP spectrum of the concentrated diamond at 6.9 T (pink arrow in Figure 3a and pink trace in Figure S2a in the SI), in comparison to *t*_*buildup*_ of 15 min and a *β*_*buildup*_ of 1, for the dilute diamond maximal enhancement position at 6.9 T, which is much longer, as depicted in Figure 3b (light blue trace in Figure S2b). The CE mechanism in this sample is enabled by the presence of the clustered P1 centers that allow for fulfilling the CE condition by “filling the gaps” in the EPR line between the sharp peaks of the isolated P1 centers.

**Figure 3:**
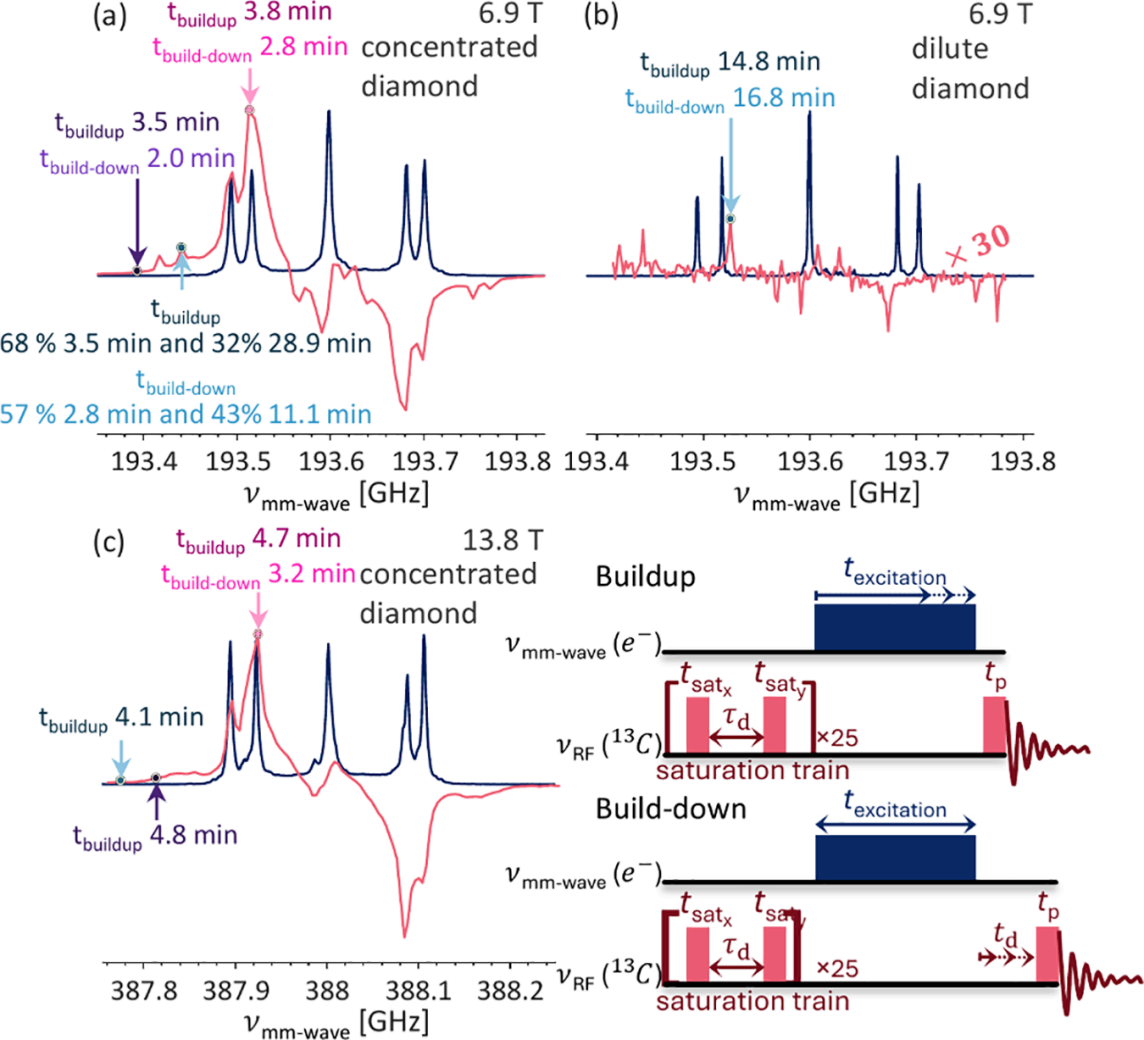
DNP buildup and build-down times for the (a and c) concentrated and (b) dilute diamond samples at (a and b) 6.9 T and (c) 13.8 T. The spectral positions of each measurement are indicated by the relevant arrow. The pink, light blue, and purple arrows probe the enhancement from coupled P1 centers with on-resonance irradiation, from both P1 populations with off-resonance irradiation, and from coupled P1 centers with off-resonance irradiation, respectively. The pulse sequences used for both experiments are shown in the inset, with the single-headed arrows indicating the varied parameter in each experiment.

All buildup times, except at 193.441 GHz for the concentrated diamond, were fitted using the stretched exponential function to facilitate their comparison and are summarized in Figure 3 and Table 1. The 95% confidence intervals for the fitting values are listed in Table 1.

**Table 1:** DNP Buildup and Build-Down Times for the Concentrated and Dilute Diamond Samples at 6.9 and 13.8 T

	concentrated diamond				dilute diamond
	coupled P1 CE-DNP	isolated P1 SE-DNP, coupled P1 iCE-DNP		coupled P1 iCE-DNP	SE-DNP
6.9 T					
frequency (GHz)	193.513	193.441	193.413	193.525	193.513
*t*_buildup_ (min)	3.8 ± 0.2	slow component (32 ± 3%)29 ± 4	fast component (68 ± 3%): 3.5 (fixed)	3.5 ± 0.7	15 ± 3
β_buildup_	0.80 ± 0.05	1	0.90 (fixed)	0.90 ± 0.23	1 ± 0
*t*_build‑down_ (min)	2.8 ± 0.2	slow component (43 ± 10%): 11 ± 3	fast component (57 ± 10%): 2.8 (fixed)	2.0 ± 1.8	17 ± 3
β_build‑down_	0.90 ± 0.05	1 (fixed)	0.90 (fixed)	0.6 ± 0.4	1 ± 0
13.8 T					
frequency (GHz)	387.922	387.775		387.811	
*t*_buildup_ (min)	4.7 ± 0.3	4.1 ± 1.9		4.8 ± 1.8	
β_buildup_	0.76 ± 0.06	0.66 ± 0.22		0.72 ± 0.23	
*t*_build‑down_ (min)	3.2 ± 0.2				
β_build‑down_	0.86 ± 0.05				

Despite CE being the dominant mechanism in the concentrated diamond, it is still interesting to evaluate to what extent SE plays a role in this sample. The SE mechanism is so universal that we expect it to at least somewhat contribute. A support for this hypothesis is that sharp features are observed in the DNP spectra of the concentrated diamond at 6.9 T for all frequencies where the SE mechanism is expected. Similar signals are visible in the magnified 13.8 T DNP spectrum in Figure 2c. They are marked with pink-blue bars in Figure 2a,c. In addition to the sharp signals, there is a significant DNP enhancement that is observed outside of the EPR line at *v*_*mm*_ _*wave*_ below 193.46 GHz or above 193.73 GHz, marked in gray in Figures 2a and c, that cannot come from CE or tCE, leaving the SE and iCE as possible candidates.

We now try to distinguish between the two mechanisms based on the buildup rates (Figure 3a). For this, we measured the buildup times at the sharp line that lies outside the EPR spectrum at 193.441 GHz as well as on the underlying broad feature at 193.413 GHz. For the latter, we observe a fast buildup time, *t*_*buildup*_ of 3.5 min, and *β*_*buildup*_ of 0.9 (purple arrow in Figure 3a and purple trace in Figure S2a). This rate is similar to the one observed for the maximum enhancement position, suggesting a CE-based mechanism, which, for irradiation outside the EPR line, can only come from the iCE. In this case, iCE would originate from excitation of the forbidden transitions, followed by spectral diffusion leading to partial saturations of the electrons participating in the CE mechanism.^27,28^ In contrast, the buildup curve acquired on the sharp feature at 193.441 GHz cannot be fitted by a single or stretched exponential, but rather the buildup curve is composed of two contributions $A_{fast}\cdot e^{-{\left(\frac{x}{t_{fast}}\right)}^{\beta_{fast}}}+A_{slow}\cdot e^{-\left(\frac{x}{t_{slow}}\right)}$ (light blue arrow in Figure 3a and light blue trace in Figure S2a). The first component was fixed on *t*_*buildup*_ of 3.5 min and *β*_*buildup*_ of 0.9 as received for the iCE contribution. Almost identical values were observed with a double exponential function with all parameters free (a comparison of the two can be seen in the Supporting Information). The second component has a much slower time constant of 29 min, with the slower component having the lower weight of the two, with only 32% weight, relative to the 68% of the fast component (Figure 2a and Table 1). We assign the second contribution to the SE originating from the isolated P1 centers. Unintuitively, the SE buildup time is longer in the concentrated diamond; we attribute it to the larger distribution of mm-wave efficiency, i.e., larger B_1_ inhomogeneity due to the larger sample size. This is supported by the nutation curves presented in the Supporting Information. We note that although the SE features in the concentrated diamond spectrum in Figure 2a appear less pronounced than in the dilute diamond spectrum in Figure 2b, the difference is accounted for by the differences in the excitation bandwidth and irradiation time.

To further substantiate the claim for observation of the iCE mechanism, we performed an electron–electron double resonance (ELDOR) experiment where we probe eSD within the coupled P1 centers originating from the forbidden EPR transitions. This experiment allows to probe the cross-talk between electron spins with different resonance frequencies. The pulse sequence is shown at the top of Figure 4. The echo intensity of electrons with resonance frequency *ν*_*probe*_ is plotted versus *ν*_*pump*_. The echo intensity is normalized to the echo intensity with the pump pulse applied far off-resonance from the EPR signal, marked by a horizontal dashed line. The normalization accounts for any hardware artifacts, e.g., heating, so any change in echo intensity indicates an interaction between the electron spins at *ν*_*pump*_ and *ν*_*probe*_. The most prominent decrease in echo intensity occurs when *ν*_*pump*_ = *ν*_*probe*_, since the probed electron spins are directly saturated. Figure 4a shows three ELDOR traces measured on the concentrated diamond. The light blue and turquoise traces, with *ν*_*pump*_ at 193.492 and 193.518 GHz, probe the interaction of isolated P1 centers, while the purple trace, with *ν*_*probe*_ at 193.55 GHz, probes the coupled P1 centers. Multiple sharp peaks can be observed in all three ELDOR spectra, matching other transitions in the electron-^14^N spin system; those are irrelevant to the current discussion and will be explored in another publication. In addition, there is a broad decrease in echo intensity spanning almost half of the EPR signal width, indicating eSD between coupled and isolated P1 centers. As expected, the broad feature is more pronounced in the coupled P1 centers ELDOR spectrum. For our investigation of the off-resonance DNP enhancement, we wish to determine if this broad feature extends beyond the spectral position of the EPR signal, marked with vertical dashed lines at 193.447 and 193.753 GHz. Outside the EPR spectrum, the eSD can only originate from irradiation of ZQ and DQ transitions, indicating a polarization transfer between the electron spins, which originates with the excitation of those forbidden transitions. This is a requirement for the iCE outside the EPR line. As expected for the effect originating from excitation of the forbidden transitions, this effect is small and requires magnification of the ELDOR spectra to be visible, as shown in the insets at the top of Figure 4. Still, it is clearly visible for all three ELDOR traces, for frequencies below 193.447 GHz at the position marked with a magenta asterisk, reinforcing our hypothesis that iCE occurs in the presence of coupled P1 centers.

**Figure 4:**
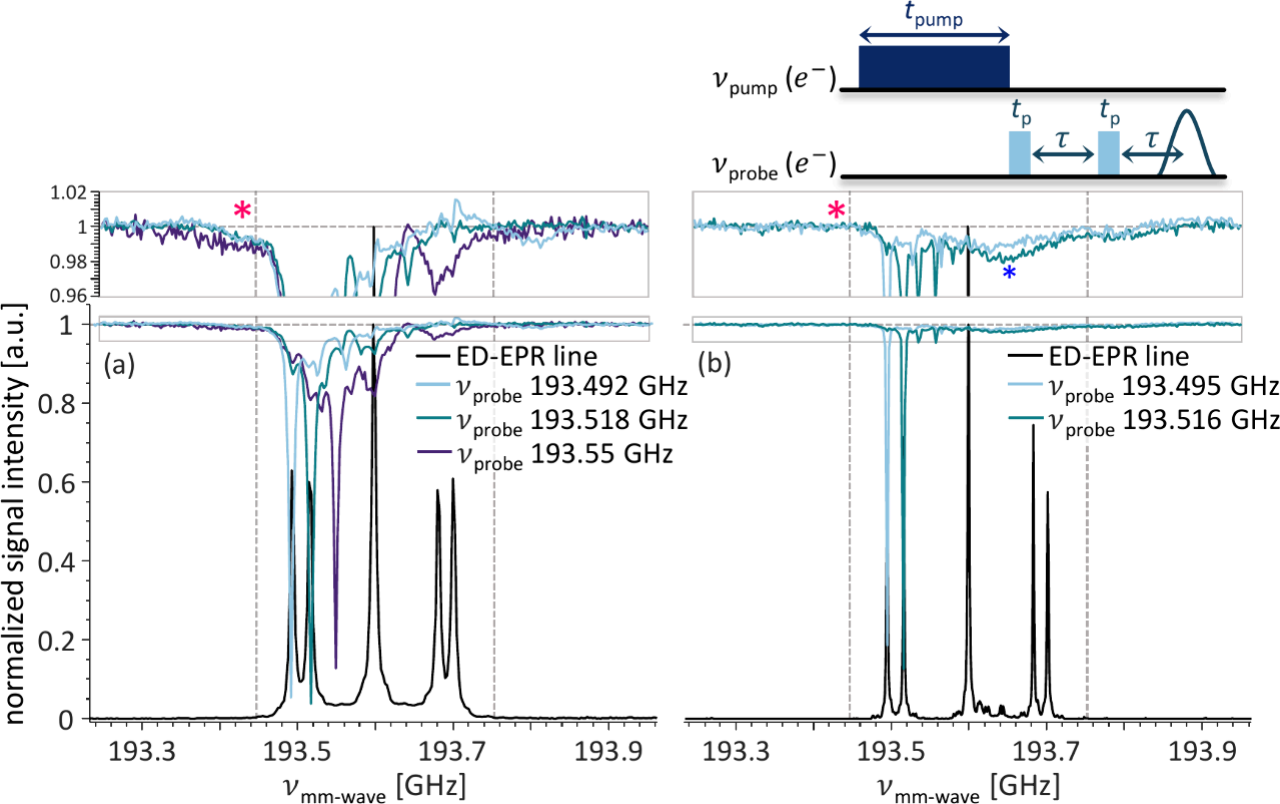
Overlays of the ED-EPR spectrum, in black, and ELDOR spectra of the (a) concentrated and (b) dilute diamond samples acquired at 6.9 T and room temperature. A close-up of the ELDOR trace baseline is shown above the figure, and the pulse sequence used is shown above. The light blue and turquoise traces probe isolated P1 centers, while the purple, only shown in panel a, probes coupled P1 centers. The normalized echo intensity, for each trace, is marked by a horizontal dashed line, and the vertical dashed lines at 193.447 and 193.753 GHz indicate the spectral position of the EPR signal. The magenta asterisks indicate the expected positions for the cross-talk required for the iCE mechanism. The blue asterisk in panel b is due to interaction with a different paramagnetic species, which is irrelevant to the P1-DNP.

In the ELDOR spectra of the dilute diamond, shown in Figure 4b, we see a different picture. Instead of the crowded ELDOR spectrum with multiple broad and narrow features, there are only four sharp ELDOR signals. As usual, the most prominent feature appears at *ν*_*probe*_ = *ν*_*pump*_ when the spins are directly saturated. The others are due to other transitions in the electron-^14^N spin system, similar to what is observed for the concentrated diamond. Notably, no broad feature is observed at the lower frequencies outside the EPR line (magenta asterisk), highlighting the absence of iCE in this sample. The broad feature observed around 193.645 GHz, marked with a blue asterisk, originates from interaction with an overlapping fast-relaxing species, which can be observed by continuous-wave EPR (data not shown). The g-factor difference between the fast relaxing species and the P1 centers, Δg of 0.00045, matches the one reported by Susumu Takahashi for a negatively charged monovacancy V^–^.^34^ These species are unrelated to the P1 centers and do not influence P1-DNP, since no broad or asymmetric hyperpolarization is observed in the DNP sweep.

Based on the DNP and ELDOR spectra, we assign the majority of the DNP enhancement in the concentrated diamond to originate from the CE and iCE mechanisms. Since no asymmetry is observed in the DNP line shape at both fields and the ELDOR spectra show only a small difference in saturation efficiency between the P1 populations, which is a prerequisite for the tCE, we conclude that the tCE does not significantly contribute.

Further substantiation of this conclusion comes from the DNP sweep at 13.8 T of the concentrated diamond, which appears to be almost devoid of all sharp features at the expected SE positions, but these are visible only under magnification, marked with pink-blue bars (Figure 2c) indicating the SE mechanism has only a small contribution to the DNP enhancement at this field. Another corroboration of this conclusion can be found in the DNP buildup times shown in Figure 3c, which were measured at similar positions as in the 6.9 T results (Figure 3a) namely at the maximum enhancement, and two positions outside the EPR line on and off resonance of the isolated P1 SE position, with all three rates now being similar, ranging from 4.1 to 4.8 min and a stretching factor of 0.66–0.76 (Figures 3c and S2e and Table 1). This indicates that at 13.8 T, the CE and iCE are the dominant contributors to the DNP enhancement, as expected for such a high field and using a relatively low power (∼90 mW) solid-state mm-wave source.

After establishing that at 6.9 T two DNP mechanisms contribute to the DNP enhancement, we investigated whether the SE polarizes the same pool of nuclei as the CE. To this end, we measured the time it takes for the ^13^C hyperpolarization to return to thermal equilibrium. This nuclear relaxation time is characteristic of the nuclear environment, as, at least in diamonds with a high concentration of impurities, the nearby paramagnetic defects govern it. The results are shown in Figure 3 and Table 1. For the ^13^C NMR spectra acquired at the maximum enhancement position, the decay time is 2.8 min with 0.9 as the stretching coefficient (pink arrow in Figure 4a and pink trace in Figure S2c), while when the same experiment was repeated at the SE peak, at 193.441 GHz, the decay time was 11 min for the slow rate with the fast decay rate fixed at 2.8 min with a 0.9 stretching coefficient (light blue arrow in Figure 3a and light blue trace in Figure S2c). Since the buildup time at this position required a biexponential fit, the build-down was fitted in the same manner. The two decay rates indicate two different pools of ^13^C nuclei, one polarized via the CE and the other via SE DNP mechanisms. To substantiate this conclusion, we measured the decay rate at 193.413 MHz, away from the SE peaks where we hypothesized that the iCE DNP occurs (purple arrow in Figure 2a and purple trace in Figure S2c). If our hypothesis is correct, we expect a similar decay constant as for the maximum enhancement, where the CE is the dominant mechanism, since iCE should polarize the same ^13^C nuclei as the CE. In agreement with our assumption, the decay time of 2.0 min is similar to the one observed for the CE position and is thus consistent with our model that the broad background under the SE peaks is a result of the iCE mechanisms, thus polarizing the same nuclei as CE does.

To conclude, using two single-crystal diamonds with and without P1 clusters, we showed experimentally, for the first time, that P1-DNP efficiency at 6.9 and 13.8 T in the absence of coupled P1 centers is very low and originates solely from the SE mechanism. Using the DNP buildup rate, ED-EPR, and ELDOR experiments we showed that the presence of coupled P1 centers enables other DNP mechanisms, such as the CE and the iCE, which was not considered before, which are much more efficient at these fields and are responsible for the majority of the observed DNP effect in these samples, with a recorded enhancement factor of 540 ± 180 at 6.9 T. Interestingly, in the concentrated sample, using hyperpolarization decay rates, we observe spatial regions devoid of P1 center clusters where the enhancement originates from SE alone. This shows that the commercial HPHT diamonds have an inhomogeneous distribution of P1 centers. While in more concentrated regions, isolated P1 centers coexist with clustered P 1s, in other, more dilute regions, the isolated P1 centers are truly isolated and do not have nearby clusters to interact with. Since the Larmor frequency of the ^13^C nuclei is indistinguishable in both regimes, this conclusion was achieved only by the ability to selectively enhance different regimes in the diamond based on the electron frequency used in the DNP experiment. This shows that DNP experiments, along with the supporting EPR spectra, can also be used to characterize the spatial distribution of paramagnetic defects.

## Materials and Methods

The concentrated diamond is a 3.2 × 3.2 × 1.1 mm HPHT diamond single crystal with a uniform yellow color polished to the [100] face. It was purchased from Element 6. The diamond was placed close to the [111] orientation for the measurement to simplify the spectrum. According to the manufacturer, the diamond has a boron concentration below 0.1 ppm and a nitrogen concentration below 200 ppm. The P1 center concentration is ∼ 20 ppm as determined by spin counting. We attribute the discrepancy between the general nitrogen concentration stated by the manufacturer and the result of our spin count measurement to the presence of nitrogen containing diamagnetic and paramagnetic species besides P1 centers.^13,35^

The dilute diamond is a 3 × 3 × 0.5 mm CVD single crystal with a uniform pink color polished to the [100] face. It has a nitrogen concentration of ∼ 13 ppm and a P1 center concentration of ∼ 3.5 ppm as determined by spin counting. The diamond has undergone irradiation and annealing for the generation of NV centers and contains both NV centers and other vacancy related defects.

The spin counting was performed on a CW X-band Bruker Elexsys E500 spectrometer.

All measurements presented were performed at room temperature using a home-built DNP/EPR spectrometer operating at 6.9 and 13.8 T. The design of the spectrometer^23^ and pulsed-EPR capabilities^8^ were described in previous publications.

The simulations were performed using the MATLAB EasySpin toolbox.^36^

The Supporting Information provides a detailed description of the experimental methods and the parameters used in the simulations.

## Supplementary Material



## Notes

13.8 T EPR spectra and simulations, 6.9 and 13.8 T buildup and build-down traces, 6.9 T ELDOR results, and experimental parameters and pulse sequences (PDF)
