# Supplementary material for: Clustering and Heterogeneous P1 Distributions in Diamond Govern DNP Mechanisms at 6.9 and 13.8 T
Source: J Phys Chem Lett. 2025 Oct 13;16(42):10952–60. doi: 10.1021/acs.jpclett.5c01809 (PMC12557381; doi:10.1021/acs.jpclett.5c01809)
Supplement: Supplementary file 1 [file jz5c01809_si_001.pdf]

Supporting Information for: Clustering and Heterogeneous P1 Distributions in Diamond Govern DNP Mechanisms at 6.9 and 13.8 T

Orit Nir-Arad[1], David H. Shlomi[1], Raj K. Chaklashiya[2,3], Nurit Manukovsky[1], and Ilia Kaminker[*1].

[1]*School of Chemistry, Faculty of Exact Sciences, Tel Aviv University, Tel Aviv 6997801, Israel.*

[2]*Department of Chemistry, Northwestern University, 633 Clark Street, Evanston, Illinois 60208, United States.*

[3]*Materials Department, University of California, Santa Barbara, Santa Barbara, California 93106, United States.*

** - Corresponding Author (iliakam@tauex.tau.ac.il)*

6.9 and 13.8 T EPR Spectra and Simulations

Figures S1a, d, f, and i reproduce the EPR line simulation from Figures 1b, d, f, and h in the main text. They show the contribution of the isolated and coupled P1 populations, in light blue and purple, respectively, for the concentrated (S1a and f) and dilute (S1d and i) diamonds at 6.9 (S1a and d) and 13.8 T (S1f and i).

To illustrate the expected contribution of each P1 population via the SE DNP mechanism, Figures S1b-c, e, g-h, and j show the predicted positions of the SE DNP lines from the isolated and coupled populations. The figures were obtained by shifting the EPR line simulation in Figures S1a, d, f, and i by -^{13}C Larmor frequency, and shifting it by +^{13}C and inverting it to obtain the expected positions of the positive and negative SE-DNP lines, respectively. Since the SE DNP line shape reflects the EPR line shape, the contribution of each population should be distinctly visible even without explicit simulations of the DNP sweep.

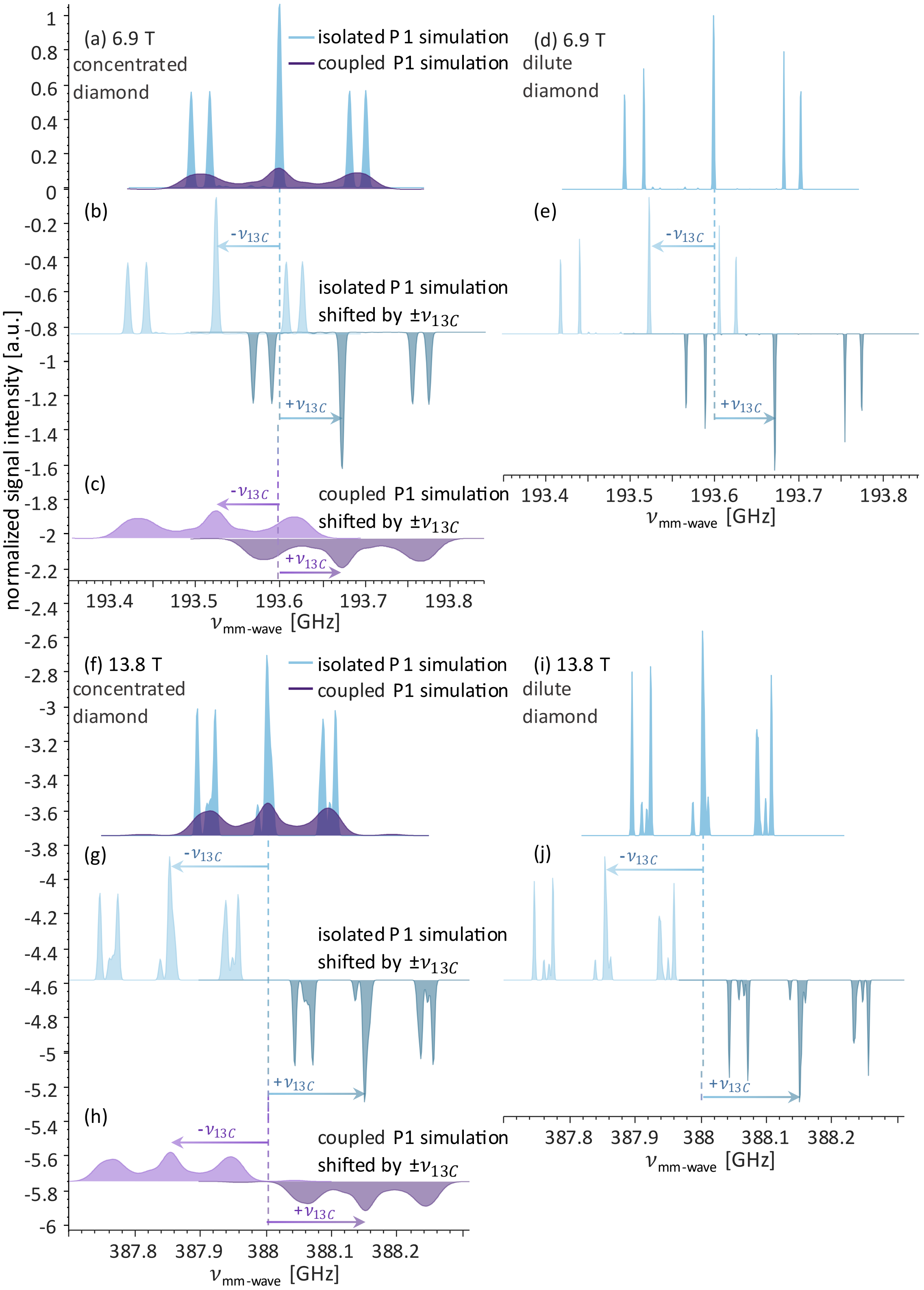

Figure S1. Simulated EPR spectra of isolated and coupled P1 centers, in light blue and purple, respectively, for the (a-c, f-h) concentrated and (d-e, i-j) dilute diamonds at (a-e) 6.9 and (f-j) 13.8 T. The expected positions of the DQ and ZQ forbidden transitions of each population are shown at the bottom, for the (b-c, g-h) concentrated and (e, j) dilute diamonds.

6.9 and 13.8 T Buildup and Build-Down Traces

To ease their comparison, all buildup and build-down traces, except at 193.411 GHz, were fitted using a stretched exponential model $e^{-(x/t_{\mathrm{buildup}})^{\beta_{\mathrm{buildup}}}}$. The experimental traces, along with the final fitting curves, are shown in Figure S2. A summary of the fitting values is shown in the main

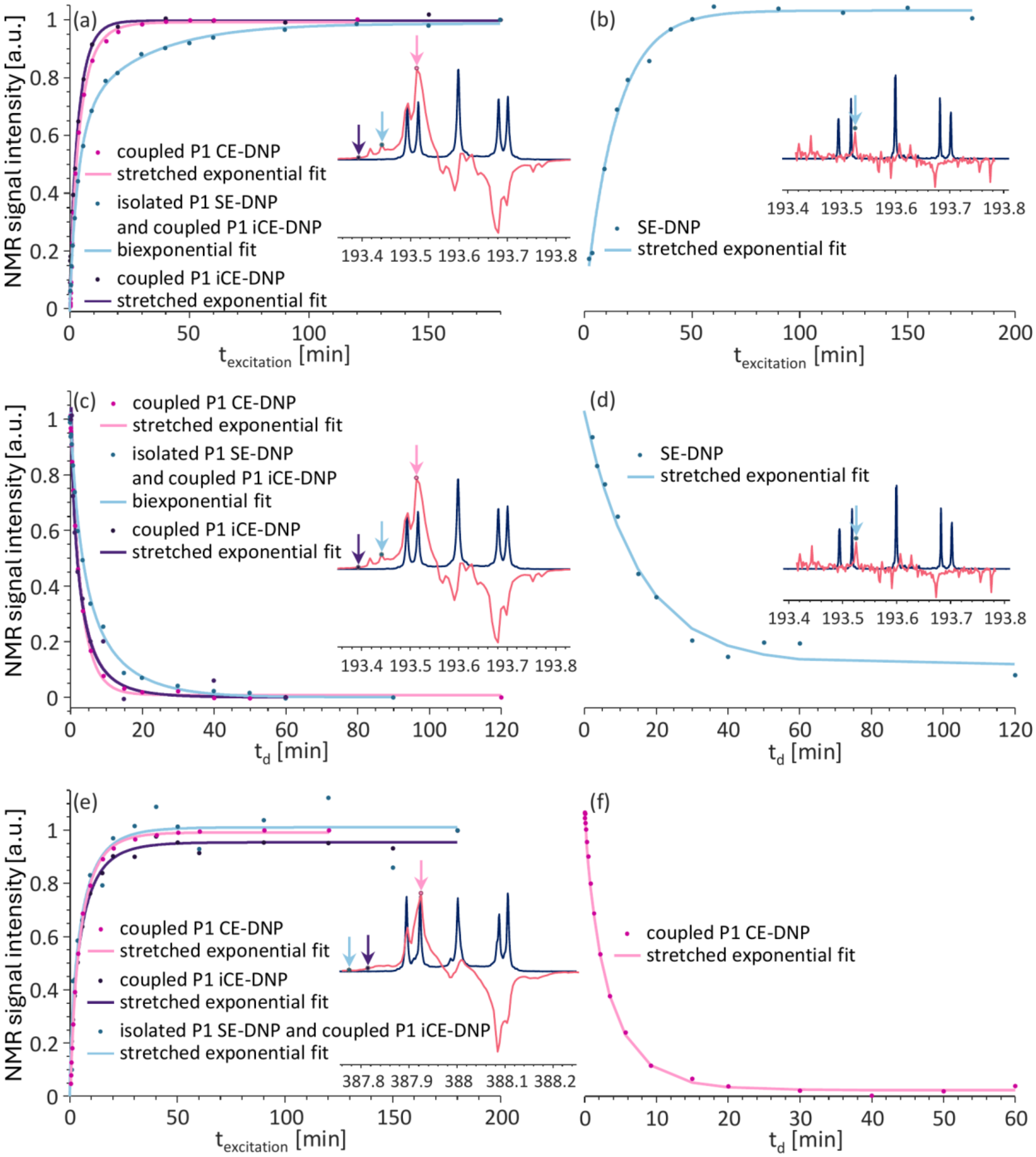

Figure S2. DNP buildup and build-down curves for the (a, c, e, and f) concentrated and (b and d) dilute diamond samples at (a-d) 6.9 and (e-f) 13.8 T, along with the exponential fits. The spectral positions where each curve was measured are shown in the insets.

The pink, light blue, and purple traces probe the enhancement from coupled P1 centers with on-resonance irradiation, from both P1 populations with off-resonance irradiation, and from coupled P1 centers with off-resonance irradiation, respectively.

text in Figure 3 and Table 1. Table 1 also includes the 95 % confidence intervals. The only exception is the buildup and build-down traces probing the isolated P1 centers SE position of the concentrated diamond at 6.9 T. Figure S3 shows the (a) buildup and (b) build-down curves at the aforementioned position with monoexponential, stretched exponential, and biexponential fitting models. Since both monoexponential and stretched exponential (shown in blue and red, respectively, in Figure S3a) do not return a satisfactory fit to the buildup curve, a biexponential fitting model was used. The fast component of a fully free biexponential fit (green trace in Figure S3a) was comparable to the buildup time at the other two positions of the concentrated diamond, so a biexponential fit, with the fast component fixed with buildup time t_{buildup} of 3.49 minutes and a stretching factor β_{buildup} of 0.9 was used for the final fit. Since two components were used for the buildup curve, two components are also expected for the build-down curve, which was similarly fitted using a biexponential fitting model with the fast component fixed on the values returned for the other positions, with decay time $t_{\mathrm{build-down}}$ of 2.8 minutes and a stretching factor $\beta_{\mathrm{build-down}}$ of 0.9.

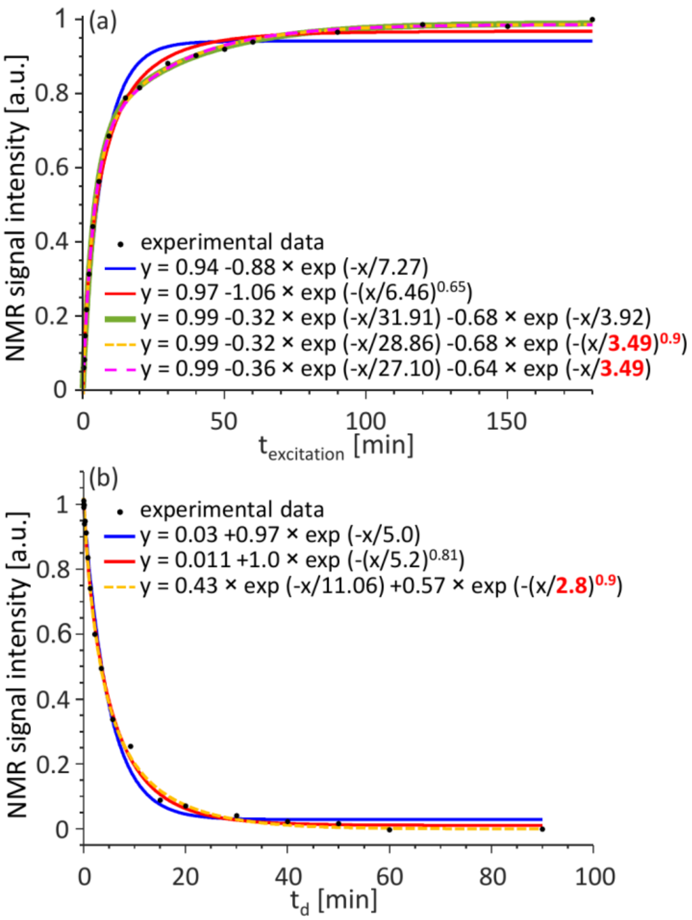

Figure S3. DNP buildup and build-down curve for the concentrated diamond samples at 193.441 GHz and 6.9 T, shown in black, along with the exponential fits, using either a monoexponential, stretched exponential, or biexponential model. The fitting values are shown in the legend, with the fixed values marked in red.

6.9 T Nutation Traces

Interestingly, the SE buildup time is longer in the concentrated diamond than in the dilute one, with 29 minutes compared to 15 minutes. Based on the diamond's size and P1 concentration, one would expect a similar or faster buildup time for the concentrated diamond. We attribute the observed difference to the differences in the mm-wave irradiation strength to which SE DNP is very sensitive. Due to its larger size, the B_1 distribution is larger in the concentrated diamond. This is confirmed by the nutation experiment recorded for each diamond, as shown in Figure S4 for the (a) concentrated and (b) dilute diamonds. It is clear that the oscillations decay faster for the concentrated diamond, indicating a larger B_1 distribution. This leads to parts of the diamond that experience a lower mm-wave power having longer SE buildup times.

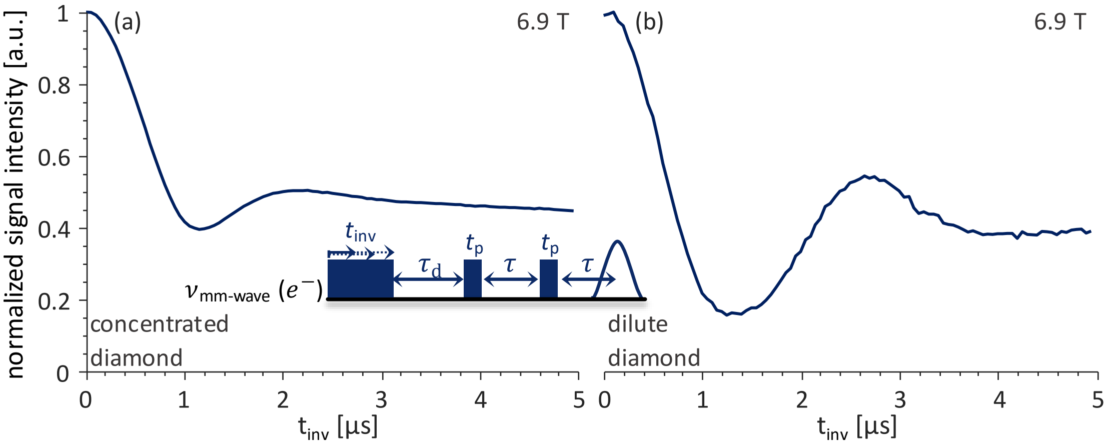

Figure S4. Nutation traces for the concentrated and dilute diamond samples at 6.9 T. The pulse sequence is shown in the inset.

Experimental Methods

The ED-EPR spectra were acquired using the sequence shown in Figure S5a with the following 16-step phase cycle, used to account for mixer imperfections.
$\phi_{\text{p1}} = [0°_4, 90°_4, 180°_4, 270°_4]$,
$\phi_{\text{p2}} = [0°, 90°, 180°, 270°]_4$,
and $\phi_{\text{detection}} = \phi_{\text{p1}} - 2\phi_{\text{p2}}$.
The parameters used for each spectrum are summarized in Table S1.

The ^{13}C P1-DNP spectra shown in Figures 2, 3, and S2 and 3 were acquired using the sequence shown in Figure S5b while varying the frequency $\nu_{\text{mm-wave}}$ (DNP sweep experiments, Figure 2) or the time $t_{\text{excitation}}$ (DNP buildup spectra shown in Figures S2a-b, S2e, and S3a are summarized in Figure 3 and Table 1). The parameters used are summarized in Table S2. The ^{13}C P1-DNP build-down spectra in Figures S2c-d, S2f, and S3b and summarized in Figure 3 and Table 1, were acquired using the sequence shown in Figure S5c while varying the wait time t_{d}. The parameters used are summarized in Table S3.

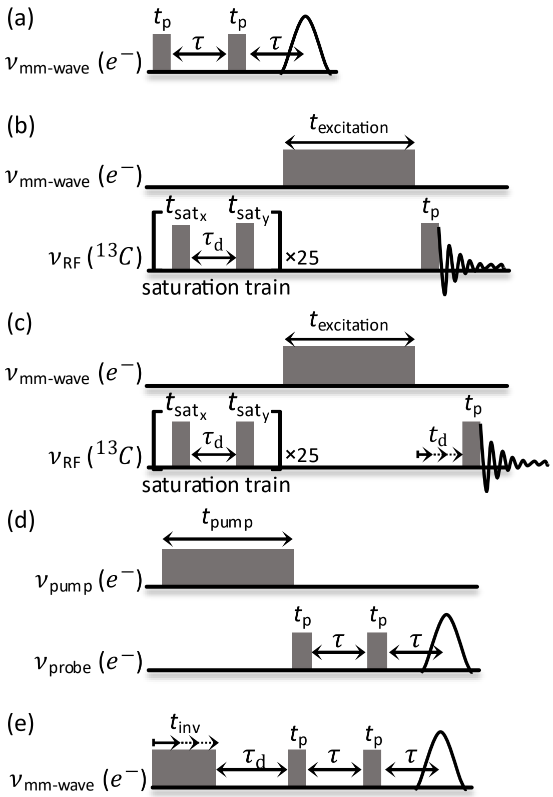

Figure S5. Pulse sequences for the (a) ED-EPR, (b and c) ^{13}C P1-DNP, (d) ELDOR, and (e) nutation experiments.

Figure	1a/2a/4a	1e/2b/4b	S1a/2c	S1e/2d
Diamond	concentrated	dilute	concentrated	dilute
Field [T]	6.9	6.9	13.8	13.8
t_{p} [μs]	0.9	1.1	1.6	1.8
τ [μs]	1.2	1	0.5	1
repetition time [ms]	3	10	2	4
averages per point	50	40	50	20

Table S1. Experimental parameters summary for the ED–EPR spectra of P1 centers in the concentrated and dilute diamonds.

Figure	2a	2b	2c	2d	3a/S2a/S3a	3b/S2b	3c/S2e
Diamond	concentrated	dilute	concentrated	dilute	concentrated	dilute	concentrated
Field [T]	6.9	6.9	13.8	13.8	6.9	6.9	13.8
$t_{\mathrm{excitation}}$ [min]	9	45	20	60	-----	-----	-----
t_{p} [μs]	6	6	20	29	6, 8, 6	6	20
t_{sat} [μs]	6	6	6	10	6, 8, 6	6	6
τ_{d} [μs]	30	30	30	20	30	30	30
total experiment time	14.1 h	5 days 19 h	2 days 8 h	>7.5 days ~½ spectrum	9.6-15.6 h	15.6 h	13.1 h

Table S2. Experimental parameters summary for the DNP sweep and buildup of the concentrated and dilute diamonds.

Figure	3a/S2c/S3b	3b/S2d	3c/S2f
Diamond	concentrated	dilute	concentrated
Field [T]	6.9	6.9	13.8
$t_{\mathrm{excitation}}$ [min]	0.5, 45, 5	20	0.5
t_{p} [μs]	6, 4, 8	6	20
t_{sat} [μs]	6	6	6
τ_{d} [μs]	30	30	30
total experiment time	7.8-33.8 h	14.6 h	5.75 h

Table S3. Experimental parameters summary for the DNP build-down of the concentrated and dilute diamonds.

The DNP enhancement factor was calculated as $\epsilon = \mathrm{I_{on}}/\mathrm{I_{off}}$, with $\mathrm{I_{on}}$ the NMR signal intensity with irradiation on-resonance with the EPR signal and $\mathrm{I_{off}}$ with irradiation far away from both the EPR signal and the SE positions. The irradiation for $\mathrm{I_{off}}$ is used to account for sample heating during the long mm-wave irradiation. The large error in the enhancement factor originates from the overlap of the diamond NMR signal and the background signal of the probe components, which is significantly stronger than that of the diamond without hyperpolarization. 24 hours of averaging were required to observe the NMR signal of the concentrated diamond without hyperpolarization. Since 45 minutes of irradiation were required to observe any appreciable enhancement for the dilute diamond at 6.9 T, measuring a similar enhancement factor would require 5 days for similar averaging, and was therefore not performed.

The ELDOR spectra in Figure 4 were acquired using the pulse sequence shown in Figure S5d, with the same phase cycle as in the ED-EPR spectra. The phase of the pulse at ν_{pump} was not cycled. The experimental parameters are summarized in Table S4.

Figure	4a	4b
Diamond	concentrated	dilute
Field [T]	6.9	6.9
t_{pump} [ms]	100	100
t_{p} [μs]	0.9	1.1
τ [μs]	1.2	0.8
repetition time [ms]	105	110
averages per point	60-130	60

Table S4. Experimental parameters summary for the ELDOR spectra of P1 centers in the concentrated and dilute diamonds.

The nutation traces in Figure S4 were acquired using the pulse sequence shown in Figure S5e. The detection sequence used the same phase cycle as in the ED-EPR spectra, while the phase of the first inversion pulse was not cycled. The experimental parameters are summarized in Table S5.

Figure	S4a	S4b
Diamond	concentrated	dilute
Field [T]	6.9	6.9
τ_{d} [μs]	10	10
t_{p} [μs]	0.9	1.1
τ [μs]	1.6	1
repetition time [ms]	2	10
averages per point	50	50

Table S5. Experimental parameters summary for the nutation traces of P1 centers in the concentrated and dilute diamonds.

The simulations were performed using the MATLAB EasySpin toolbox[1] using the *pepper* function for solid-state EPR spectra simulations. The simulations used the *g*-tensor of (2.00220, 2.00220, 2.00218),[2] the hyperfine tensor (81.3, 81.3, 114) MHz,[4] and the quadrupole coupling constant of −3.973 MHz.[4] A crystal symmetry of Fd-3m and Euler angles of (45°,54.74°,0°) were used for the molecular frame orientation relative to the crystal frame. The orientation of the crystal frame relative to the magnetic field was $(0°, 58.2°, 0°)$ and $(0°, 56.5°, -1°)$ for the concentrated and dilute diamond, respectively. The simulations of the concentrated diamond were reported previously[2,3], and used three components with the second and third combined and referred to as coupled P1 in the main text. The isolated P1 component used a Gaussian linewidth peak-to-peak of 4.52 MHz and a relative weight of 0.62. The P1 pair component accounted for dipolar-coupled P1 centers and used a Gaussian linewidth peak-to-peak of 29.9 MHz, which corresponds to a dipolar interaction of up to 47 MHz and an interspin distance of 1–2.5 nm, and a relative weight of 0.34. The last component of exchange-coupled P1 clusters used an exchange coupling of 138 MHz, which corresponds to sub-1 nm interspin distances, a Gaussian linewidth peak-to-peak of 17.5

MHz, and a relative weight of 0.04. The same parameters were used for both 6.9 and 13.8 T. For the dilute diamond simulation, only a single component was used, accounting for isolated P1 centers with a Gaussian linewidth peak-to-peak of 1 and 2.3 MHz for the 6.9 and 13.8 T simulations, respectively.

References

(1) Stoll, S.; Schweiger, A. EasySpin, a Comprehensive Software Package for Spectral Simulation and Analysis in EPR. *J. Magn. Reson.* **2006**, *178* (1), 42–55. https://doi.org/10.1016/j.jmr.2005.08.013.

(2) Nir-Arad, O.; Shlomi, D. H.; Manukovsky, N.; Laster, E.; Kaminker, I. Nitrogen Substitutions Aggregation and Clustering in Diamonds as Revealed by High-Field Electron Paramagnetic Resonance. *J. Am. Chem. Soc.* **2024**, *146* (8), 5100–5107. https://doi.org/10.1021/jacs.3c06739.

(3) Nir-Arad, O.; Laster, E.; Daksi, M.; Manukovsky, N.; Kaminker, I. On the Peculiar EPR Spectra of P1 Centers at High (12–20 T) Magnetic Fields. *Phys. Chem. Chem. Phys.* **2024**, *26* (43), 27633–27647. https://doi.org/10.1039/D4CP03055A.

(4) Cox, A.; Newton, M. E.; Baker, J. M. 13C, 14N and 15N ENDOR Measurements on the Single Substitutional Nitrogen Centre (P1) in Diamond. *J. Phys. Condens. Matter* **1994**, *6* (2), 551. https://doi.org/10.1088/0953-8984/6/2/025.
